# Novel composite based on silicone rubber and a nano mixture of SnO_2,_ Bi_2_O_3,_ and CdO for gamma radiation protection

**DOI:** 10.1038/s41598-024-51965-0

**Published:** 2024-01-18

**Authors:** Ahmed M. El-Khatib, Kareman Zard, Mahmoud I. Abbas, Mona M. Gouda

**Affiliations:** 1 Physics Department, Faculty of Science, Alexandria University, Alexandria, 21511 Egypt; 2 Medical Physics and Radiotherapy Department, Alexandria Ayadi Almostakbal Oncology Hospital, Alexandria, Egypt

**Keywords:** Nanoscience and technology, Physics

## Abstract

Recently, there has been a surge of interest in the application of radiation-shielding materials. One promising research avenue involves using free-lead metal oxides/polymer composites, which have been studied for their radiation shielding and characterization properties. This study reinforced the dimethylpolysiloxane (silicone rubber) composites with micro- and nano-sized particles of tin oxide, cadmium oxide, and bismuth oxide as additive materials. The composites were tested with 20 and 50 weight fractions, and their attenuation coefficients were measured using a NaI(TI) detector at gamma-ray energies ranging from 59.54 to 1408.01 keV. Also, the thermal and mechanical properties of the composites were observed and compared with those of free silicone rubber. The results showed that the 50% nano metal oxide/SR composites exhibited better thermal stability and attenuation properties than the other composites, also possessing unique attributes such as lightweight composition and exceptional flexibility. Consequently, this composite material holds immense potential for safeguarding vital organs, including the eyes and gonads, during radiological diagnosis or treatment procedures. Its exceptional ability to absorb a significant portion of incident rays makes it an invaluable asset in the field of radiation protection.

## Introduction

The recent development of medical technology facilities, such as radiation surgery, radiation diagnosis, and radiotherapy. The radiation employees and patients who are exposed to radiation utilize appropriate protection equipment. When living organisms are exposed to low amounts of background radiation, there is very little overall effect on the organism. When radiation exposure in larger amounts occurs, even if it is for a short time, damage, radiation poisoning, and even death can occur. The scientific community has not reached a consensus on the effects of very low doses of radiation, as described by the Radiation Answers Organization^1^. In radiation therapy, high energy ionizing radiation “up to 4 MeV and up to 6 MV” is a common protocol utilized to control and destroy malignant growth as the main part of cancer treatment. Usually, high-density lead aprons are used to protect against any type of radiation in radiotherapy and nuclear radiation. Although lead has great protection properties, it’s a highly toxic material^2–4^.

Numerous theoretical and experimental studies have investigated a variety of protection materials to attenuate unwanted radiation. They tend to design polymer nanocomposite materials that are reinforced with high Z-additives^5,6^. Polymers are macromolecules consisting of hundreds or thousands of monomers that join each other as a long chain. The main properties of polymers are low densities compared to additive metals and ease of synthesis. As polymer monomers have a large number of hydrogen atoms, they are more effective at attenuating high-energy particles such as neutrons^7–9^.

Recently, high Z-metal and metal-doped polymers have been the most common protection materials for shielding nuclear and radiation workers and patients against radiation^10–12^. Zhang et al.^13^ studied the mechanical and thermal properties of silicone rubber, which is reinforced with nano graphene platelets. They noticed improvements in their thermal and mechanical properties due to the high interface interaction between graphene platelets and SR composites**.** Guo et al.^14^ investigated the mechanical, thermal conductivity, and thermal stability properties of boron nitride/silicone rubber composites in the cases of nano and micro at different weight fractions. At 60 h of BN, thermal conductivity reaches 18 times that of free SR in two cases of nano- and micro-BN. Thermal stability increases as the BN weight fraction increases in composites. Malezadeh et al.^15^ studied the attenuation coefficients of a 10% Bi-Si matrix in micro and nano size at low X-ray energy (60–150 keV). Their findings indicate that nano-Bi composites have significantly higher attenuation than micro-Bi composites. Malezadeh et al.^16^ investigate the attenuation parameters of silicone rubber composites filled with BaSO_4_, WO_3_, and PbO in the energy range of 60–600 keV. They report that nanocomposites have higher shielding properties than micro composites.

In this work, we have used silicone rubber as a polymer reinforced with Bi_2_O_3_, CdO, and SnO_2_ as an additive material to attenuate ionizing radiation gamma rays. Linear attenuation coefficients of composites were measured in the case of micro and nano metal oxides (Bi_2_O_3_, CdO, and SnO_2_) for various weight fractions at different gamma-ray energies. We investigated the optimal difference between the measured and theatrical mass attenuation coefficients of composites. Various characterization tests have been utilized to investigate the morphology, tensile properties, and thermal properties of micro- and nano-metal oxides (Bi_2_O_3_, CdO, and SnO_2_)/silicon rubber composites.

## Materials

The silicone rubber was utilized as a polymer. By adding stiffener with a 2% weight fraction, silicone rubber transformed into a rigid composite by catalyzed reaction. The additive metal oxides (Bi_2_O_3_, CdO, and SnO_2_) have the same fraction in the mixture. The nano metal oxides were supplied by Nanotech Company, Egypt. Bi, Sn, and Cd are heavy metals characterized by their high density and high atomic number. However, the utilization of cadmium oxide (CdO) raises significant concerns due to its inherent toxicity and potential adverse environmental impact. But in radiation therapy using high-energy photon beams (E > 10 MeV), neutrons are primarily generated in the linac head through interactions known as (γ,n), where photons interact with the nuclei of high atomic number materials present in the linac head and the beam collimation system. The presence of these neutrons impacts the necessary shielding requirements within radiation therapy rooms and also leads to an increase in the out-of-field radiation dose for patients undergoing radiation therapy with high-energy photon beams. The presence of Cd will help absorb these neutrons, especially the thermal ones.

Silicone rubber is produced commercially. Enhanced production of silicone rubber involves the incorporation of nanomaterials, which unfortunately leads to an increase in costs. However, a promising solution to mitigate these expenses lies in the utilization of the ball milling method. This technique enables the transformation of bulk materials into nanoscale counterparts, thereby reducing overall production costs.

To protect the environment, it shouldn’t dispose of silicone items indiscriminately. Instead, make a better choice by sending silicone items to specialized recycling companies. Silicone rubber is a durable material and recycled many times. Also, it can send them off to your local recycling centers to get them properly recycled^17–19^.

### Synthesis of metal oxides/SR composites

The metal oxides (Bi_2_O_3_, CdO, and SnO_2_)/SR samples were prepared by mixing and molding. The silicone rubber is loaded with micro- and nano—(Bi_2_O_3_, CdO, and SnO_2_) by mixing for different weight fractions. After that, add a stiffener “vulcanizing agent” to the mixture at a concentration of 2%. To remove air bubbles from the matrix, the matrix was vacuumed for 30 min. The samples were placed in laboratory to 48 h to dry then employed in experimental steps, then mold at room temperature for 24 h to obtain metal oxides/SR samples^20,21^.

## Characterization parameters

### Scanning and transmission electron microscope

The size of the nano fillers was analyzed using a transmission electron microscope (FE-TEM) manufactured by JEOL, Japan, operating at 200 kV. The size of micro fillers was measured using a scanning electron microscope (SEM) [JSM-6010LV, JEOL]. Furthermore, the scanning electron microscope (SEM) was employed to observe the distribution of micro- and nano-sized particles (Bi_2_O_3_, CdO, and SnO_2_) within the composites’ cross-section^22–24^.

### Mechanical test

**Figure 1:**
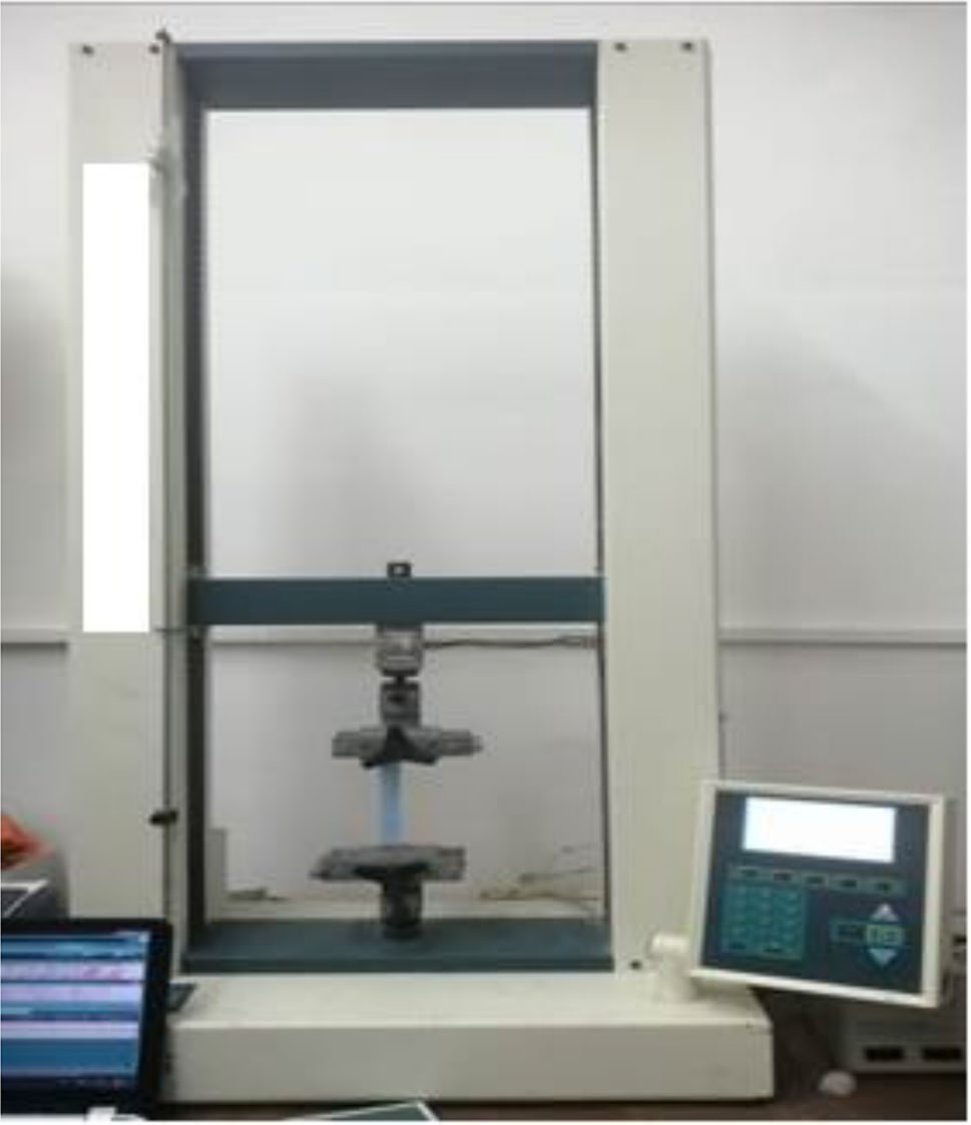
Tensile testing machine.

The tensile properties were investigated using a Tinius Olsen [H10KS] tension testing machine according to [ASTM D882-10] at room temperature, as shown in Fig. 1. The dumbbell-shaped composites were crossed at 100 mm/min^25–27^.

### Thermogravimetric analysis (TGA)

The thermal properties of composites were investigated by a TGA [SDT-Q600] machine. Changes in thermal stability of micro- and nano—(Bi_2_O_3_, CdO, and SnO_2_)/SR were tested at 10 °C/min heating rate as a function of temperature from 25 to 800 ^o^C^28–30^.

## Shielding parameters

Shielding characterization of samples was studied by five radioactive sources (^152^Eu, ^133^ Ba, ^137^Cs, ^60^Co and ^241^Am) in energies (59.54 − 80.99 − 121.78 − 244.69 − 356.01 − 661.66 − 778.9 − 964.13 − 1173.23 − 1332.5 − 1408.01) keV using a NaI “3 × 3” (TI) detector and software analyzer winTMC spectrum vision. The experimental data have been measured in the presence and absence of samples to count initial intensity and attenuated intensity. The radiation shielding parameters are explained in Table 1. Also, the experimental MAC was compared with the XCOM program “theoretical data”^31–36^.

**Table 1:** Evaluation equation of radiation shielding parameters for silicone rubber composites.

Parameter	Equation	Unit
Linear attenuation coefficient (LAC)	$\mu = \frac{ - 1}{x} ln\frac{I}{{I_{o} }}$	1/cm
Mass attenuation coefficient (MAC)	μ_m_ = $\frac{{\varvec{\mu}}}{{\varvec{\rho}}}$	cm^2^/g
Half value layer (HVL)	$HVL = \frac{LN \left( 2 \right)}{\mu }$	cm
Tenth value layer (TVL)	$TVL = \frac{{LN \left( {10} \right)}}{\mu }$	cm
Mean free path (MFP)	$\lambda = \frac{1}{\mu }$	cm
Effective atomic number (*Z*_*eff*_)	*Z*_*eff*_ = $\frac{{\Sigma_{i } w_{i} A_{i} \left[ {\frac{\mu }{\rho }} \right]_{i} }}{{\Sigma_{i } w_{i } \frac{{A_{i} }}{{z_{i} }} \left[ {\frac{\mu }{\rho }} \right]_{i} }}$	g
Deviation between the MAC of experimental and theoretical data from XCOM ($({\text{Dev \%}})$	${\text{Dev}} = \left( {\frac{{{\text{MAC}}_{{{\text{XCOM}}}} - {\text{MAC}}_{{{\text{EXP}}}} }}{{{\text{MAC}}_{{{\text{EXP}}}} }}} \right) \times 100$	%

## Results and discussion

### Scanning and transmission electron microscope

**Figure 2:**
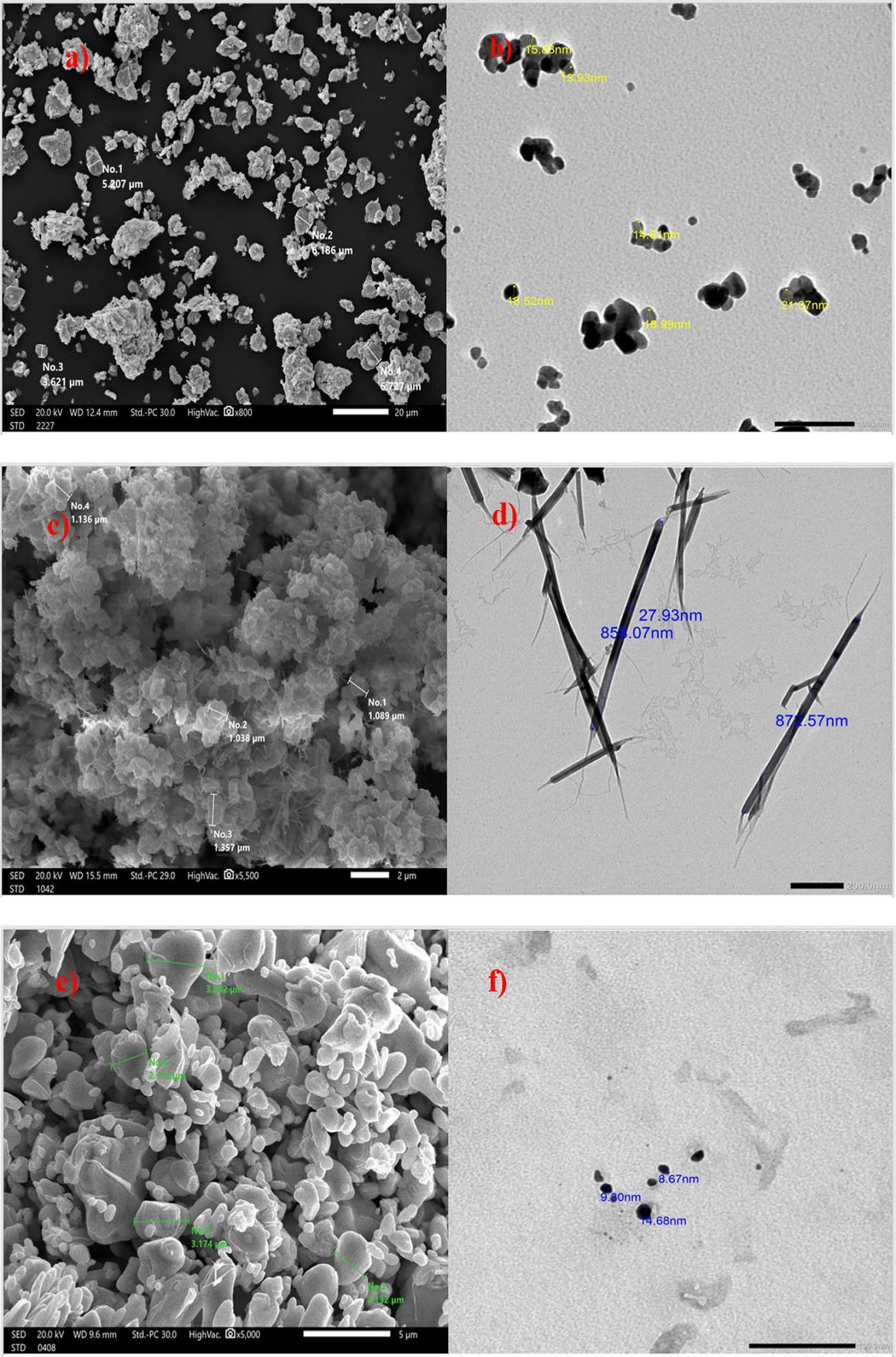
(**a**, **c**, and **e**) SEM image of micro-tin oxide particles, micro-cadmium oxide, and micro-bismuth oxide respectively. (**b**, **d**, and **f**) TEM image of nano-tin oxide particle, nano-cadmium oxide rods, and nano-bismuth oxide respectively.

Figure 2 showcases an image captured by a scanning electron microscope (SEM), displaying micro-sized particles of cadmium oxide rods, tin oxide, and bismuth oxide particles. Furthermore, it presents a transmission electron microscope (TEM) image, revealing nano-sized of these particles. The average size of micro metal oxides is in the range of 1.038–6.186 μm as shown in Fig. 2a, c, and e while Fig. 2b, d, and f show the average size of nanometal oxides in the range of 8.67–27.93 nm.

**Figure 3:**
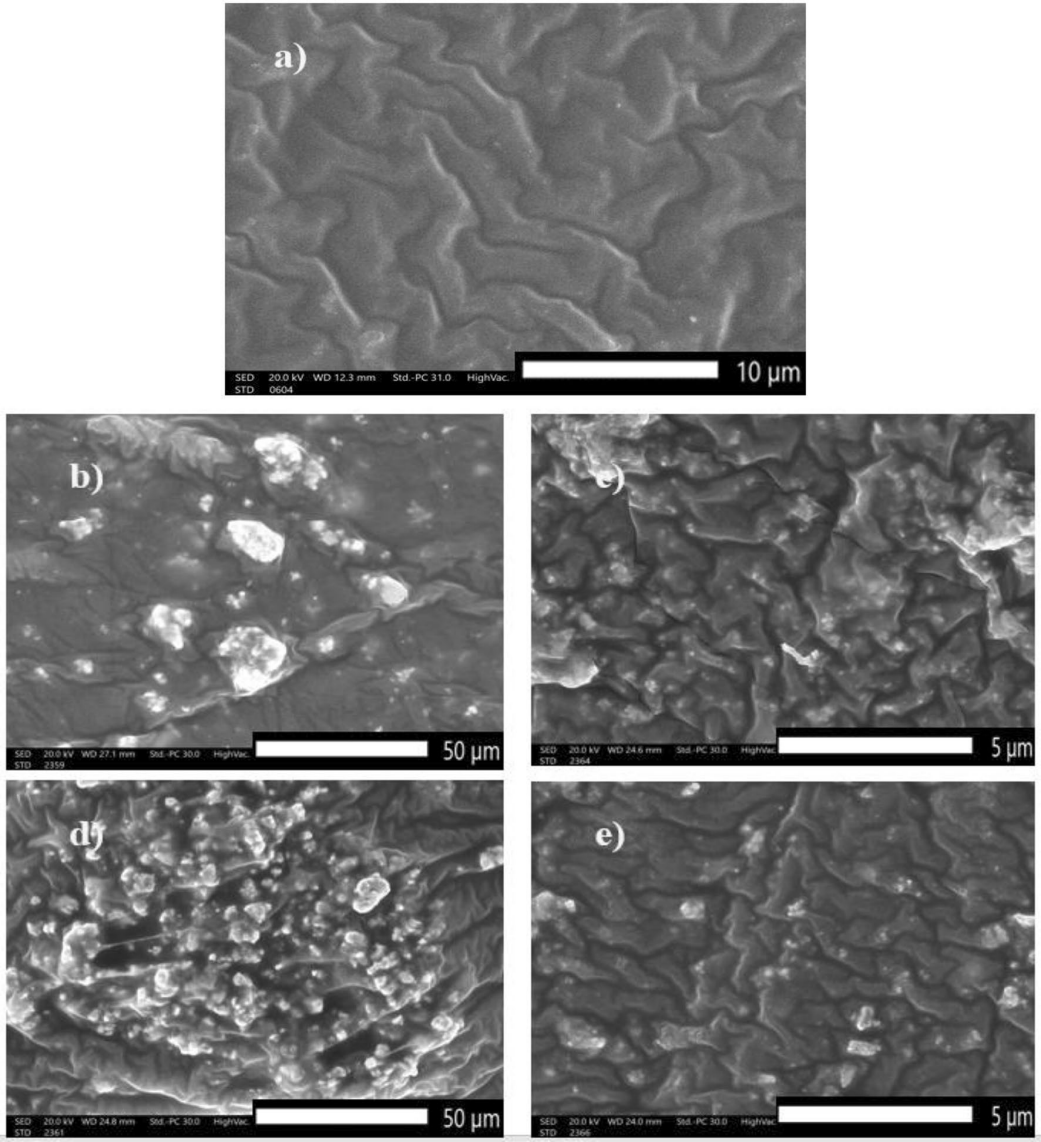
SEM image of (**a**) silicone rubber, (**b**) 20% micro Bi_2_O_3_, CdO, and SnO_2_/SR, (**c**) 20% nano Bi_2_O_3_, CdO, and SnO_2_/SR, (**d**) 50% micro Bi_2_O_3_, CdO, and SnO_2_/SR, and (**e**) 50% nano Bi_2_O_3_, CdO, and SnO_2_/SR.

Figure 3 represents the SEM micrographs of SR, micro- and nanocomposites. From Fig. 3a, the free silicone rubber cross-section has a clear and regular structure, while the reinforced silicone rubber composites have a more erratic and rough structure. but at nanocomposites, nano—(Bi_2_O_3_, CdO, and SnO_2_) were better dispersed than micro—(Bi_2_O_3_, CdO, and SnO_2_) in the silicone rubber matrix. When the additive material weight fraction increases from 20 to 50%, agglomerations of filler increase in micro-composites and interparticle distances become smaller, which affects mechanical properties.

### Mechanical results

**Figure 4:**
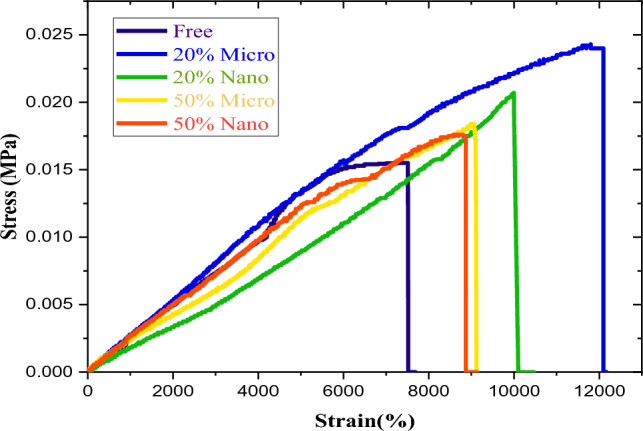
Mechanical curves of SR, 20% micro—Bi_2_O_3_, CdO, and SnO_2_/SR, 20% nano—Bi_2_O_3_, CdO, and SnO_2_/SR, 50% micro—Bi_2_O_3_, CdO, and SnO_2_/SR, and 50% nano—Bi_2_O_3_, CdO, and SnO_2_/SR composites.

Figure 4 shows stress–strain curves of free silicone rubber and metal oxides/SR composites. The additives (Bi_2_O_3_, CdO, and SnO_2_) improve the ultimate stress and tensile strength of silicone rubber composites. Moreover, with an increment in filler weight fraction, first the ultimate stress and tensile strength increase at 20% concentration and then decrease at 50% concentration because of the agglomeration of filler particles in silicone rubber, which decreases the strain of SR composites. As described in Fig. 4, tensile strength of SR composites follows the order: free-SR < 50% nano < 50% micro < 20% nano < 20% micro, where the ultimate stress of micro-SR composites is higher than that of nano-SR composites at the same concentration. At 50% micro and nano concentrations, ultimate stress and strain tend to decrease at 50%, so that the expected enhancement does not occur and tensile properties will decrease. Therefore, the limitation line for silicone rubber must be achieved at less than 50% concentration.

### Thermogravimetric analysis results

**Figure 5:**
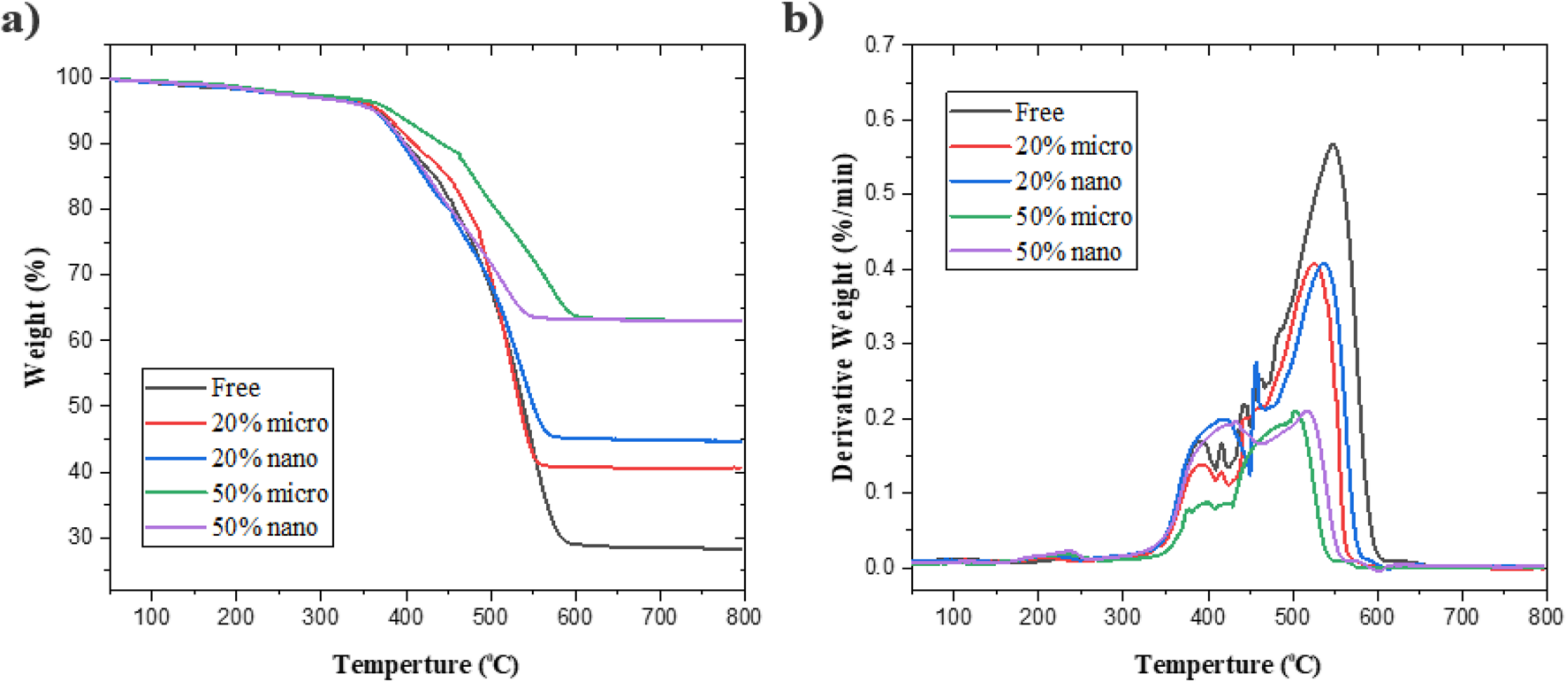
(**a**) TGA, (**b**) differential TGA of different composites.

It’s known that metal oxides have high-temperature stability. So when (Bi_2_O_3_, CdO, and SnO_2_) are added to silicone rubber composites. It has ameliorated and improved the thermal stability of composites. Figure 5 represents the TGA and differential TGA results of (Bi_2_O_3_, CdO, and SnO_2_)/SR composited. For free silicone rubber, it has thermal stability at around 300 ^o^C but after 450 ^o^C it gradually decomposes, and weight loss reaches 71.68%. By adding metal oxides, the thermal stability of silicone rubber composites is overall improved, and the weight loss percentage decreases. Moreover, thermal stability increases with increasing weight fractions of metal oxides at the micro and nanoscales. Figure 5a shows nanocomposites have lower weight loss and better thermal properties than micro composites at the same weight fraction. Figure 5b shows that the DTGA peak decreases as an additive material increases because of the presence of inorganic composites in a mixture where the specific heat capacity of metal oxides is much larger, and the heat absorption efficiency is higher.

### Shielding results

The measured result of MAC of metal oxides (Bi_2_O_3_, CdO, and SnO_2_)/SR and theoretical data utilizing XCOM software are listed in Table 2. The deviation values were estimated for each concentration to explain the acceptable harmony percentage between two values for all energies, where deviation for free silicone rubber is in the range of − 3.633 to 4.302, for 20% (Bi_2_O_3_, CdO, and SnO_2_)/SR between − 3.921 and 2.493, and for 50% (Bi_2_O_3_, CdO, and SnO_2_)/SR between − 3.099 and 2.122. This harmony indicates the validation of the experimental system.

**Table 2:** The theoretical Xcom values and the measured mass attenuation coefficients for SR, 20% and 50% micro- (Bi_2_O_3_, CdO, and SnO_2_)/SR composites.

Energy—keV	Free silicone rubber			20% (Bi_2_O_3_, CdO, and SnO_2_)/SR			50% (Bi_2_O_3_, CdO, and SnO_2_)/SR		
	MAC (measured)	MAC (XCOM)	Dev %	MAC (measured)	MAC (XCOM)	Dev %	MAC (measured)	MAC (XCOM)	Dev %
59.54	0.2186	0.2245	2.690	1.1663	1.1830	1.433	2.5578	2.6150	2.237
80.99	0.1796	0.1841	2.491	0.5698	0.5736	0.672	1.1950	1.1580	− 3.099
121.78	0.1641	0.1581	− 3.633	0.4385	0.4319	− 1.502	0.8334	0.8475	1.690
244.69	0.1531	0.1564	2.148	0.1672	0.1606	− 3.921	0.2167	0.2162	− 0.240
356.01	0.1209	0.1237	2.331	0.1175	0.1187	1.012	0.1398	0.1353	− 3.233
661.66	0.1067	0.1076	0.878	0.0817	0.0833	1.943	0.0839	0.0833	− 0.792
778.9	0.0798	0.0833	4.302	0.0747	0.0765	2.493	0.0738	0.0754	2.122
964.13	0.0750	0.0780	3.876	0.0674	0.0685	1.603	0.0659	0.0665	0.937
1173.23	0.0680	0.0699	2.785	0.0627	0.0619	− 1.369	0.0597	0.0595	− 0.409
1332.5	0.0573	0.0595	3.822	0.0577	0.0579	0.315	0.0567	0.0555	− 2.064
1408.01	0.0578	0.0578	− 0.052	0.0570	0.0562	− 1.357	0.0537	0.0539	0.503

**Figure 6:**
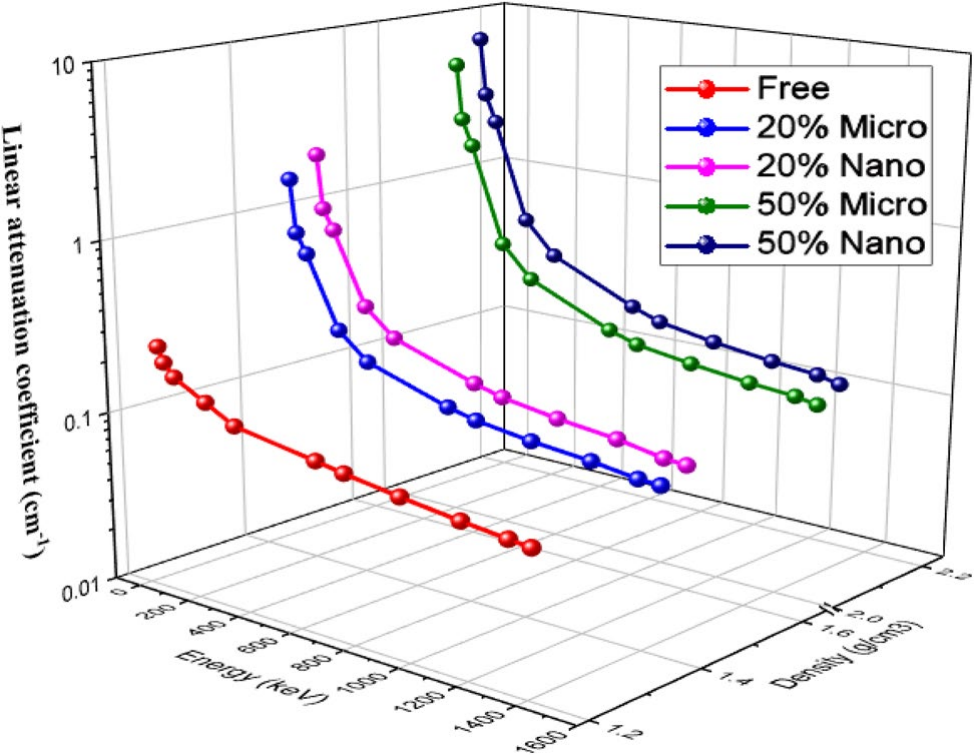
Comparison between LAC for nano- and micro—Bi_2_O_3_, CdO, and SnO_2_/SR at different energy photons at 20% and 50% weight fraction as a function of density.

Figure 6 represented the effect of additive metal oxides (Bi_2_O_3_, CdO, and SnO_2_) on the increment of LAC for free silicone rubber, 20% (Bi_2_O_3_, CdO, and SnO_2_)/SR and 50% (Bi_2_O_3_, CdO, and SnO_2_)/SR at all interested energies. The curves explain that nano (Bi_2_O_3_, CdO, and SnO_2_)/SR composites have a definite superiority of LAC over micro—(Bi_2_O_3_, CdO, and SnO_2_)/SR composites at the same concentration, as nano—(Bi_2_O_3_, CdO, and SnO_2_) particles have a more uniform distribution in the silicone rubber mixture than micro—(Bi_2_O_3_, CdO, and SnO_2_), which led to a decrease in empty space in composites. So that beam transformation in the side matrix decreases and enhances the probability of interaction between gamma ray photons and particles. Moreover, the surface-volume ratio of nano—(Bi_2_O_3_, CdO, and SnO_2_) particles is significantly higher than that of micro—(Bi_2_O_3_, CdO, and SnO_2_) particles, so the cross-section of collision between beam photons and particles increases in the case of nanoparticles. Density is a critical parameter affecting the attenuation efficiency of composites. Whereas (Bi_2_O_3_, CdO, and SnO_2_) concentrations in mixtures increase, the density of composites increases. Moreover, nano—(Bi_2_O_3_, CdO, and SnO_2_) composites have higher densities than micro—(Bi_2_O_3_, CdO, and SnO_2_) composites at the same weight fraction, so LAC increases.

**Figure 7:**
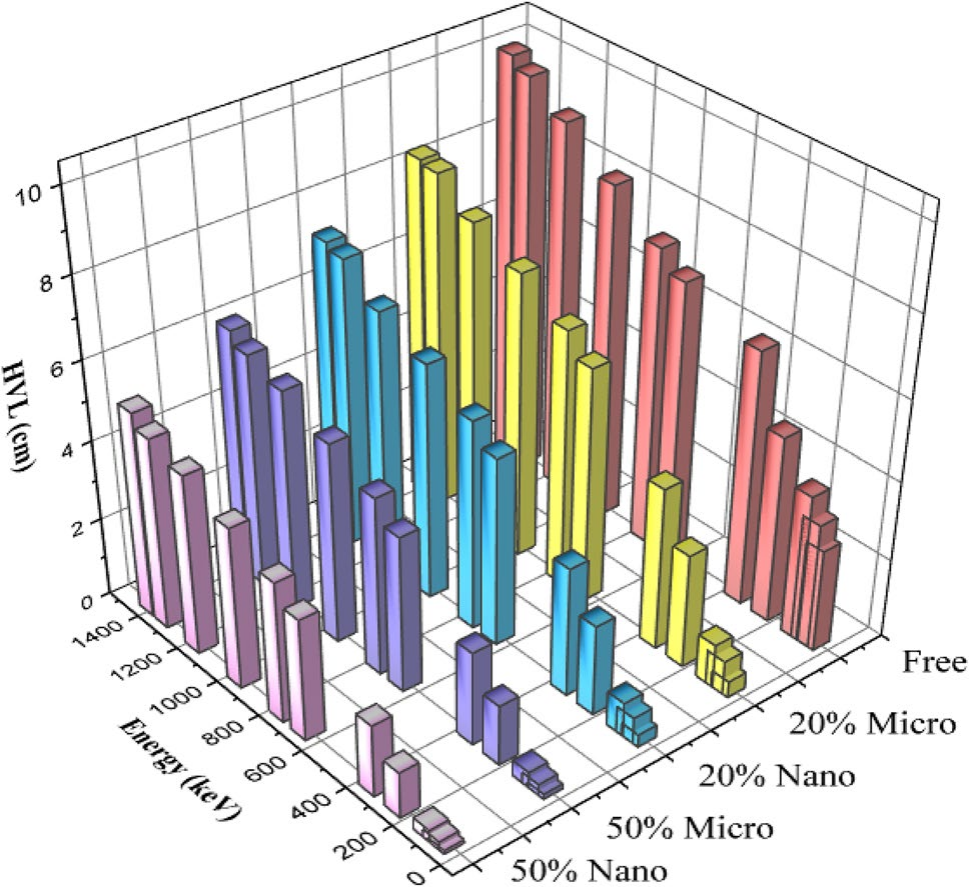
HVL of silicone rubber, micro-and nano—Bi_2_O_3_, CdO, and SnO_2_/SR for different weight fraction at different energy.

**Figure 8:**
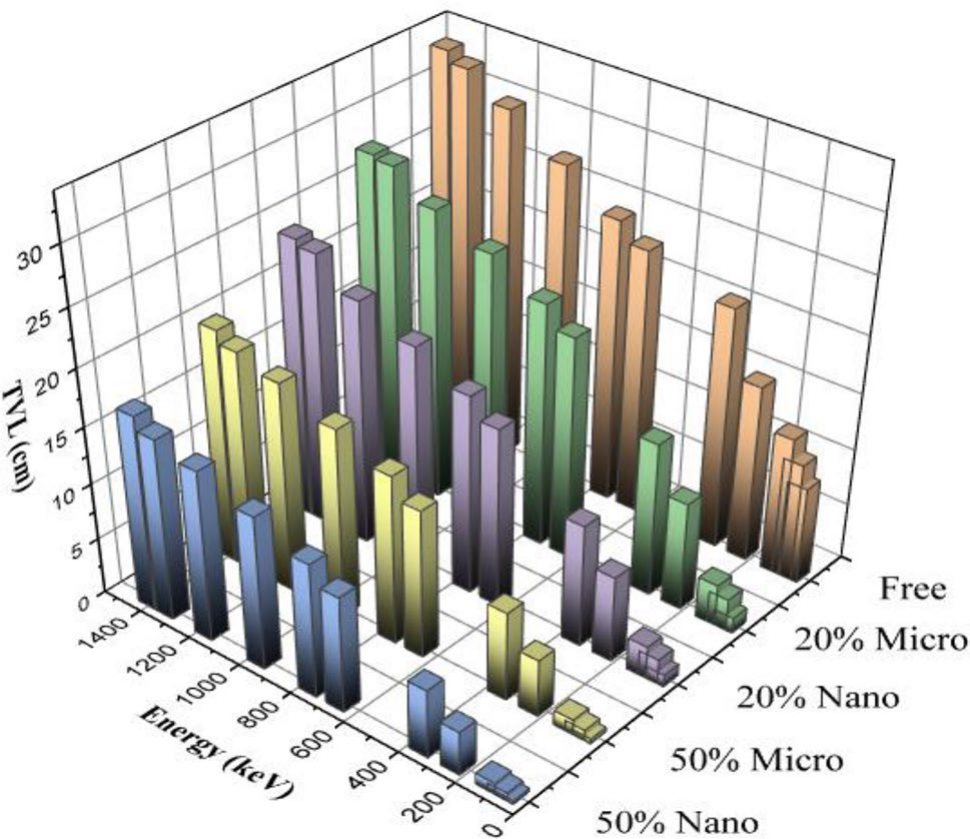
TVL of silicone rubber, micro-and nano—Bi_2_O_3_, CdO, and SnO_2_/SR for different weight fraction at different energy.

The HVL and TVL of shielding composites are the main affected factors of the gamma protection design. As seen in Figs. 7 and 8, the HVL and TVL values represented the needed thickness to attenuate half and a tenth of the energy beam, respectively. HVL and TVL values increase gradually as energy increases from 59.54 to 1408.01 keV. It can be observed that, HVL and TVL rates of nano—(Bi_2_O_3_, CdO, and SnO_2_)/SR are lower than those of micro—(Bi_2_O_3_, CdO, and SnO_2_)/SR at the same weight fraction, which means higher shielding efficiency. TVL values are a critical marker of attenuation ability. Lower HVL values of composites mean better attenuation performance.

**Figure 9:**
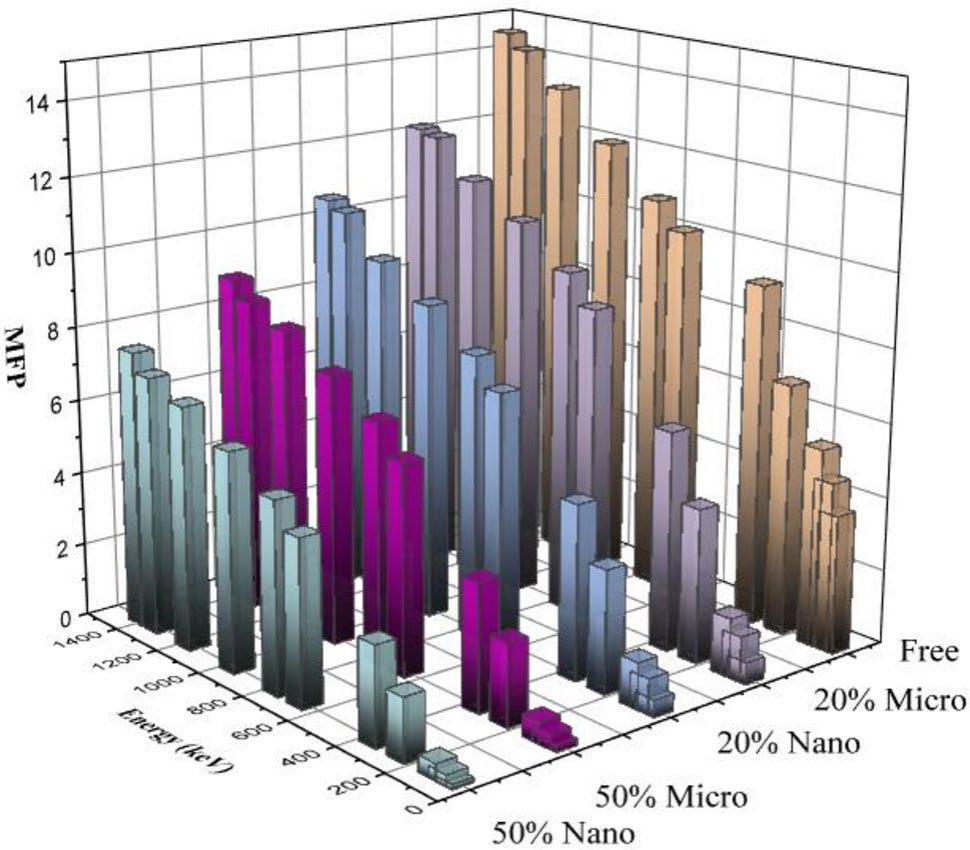
MFP of silicon rubber, micro-and nano—Bi_2_O_3_, CdO, and SnO_2_/SR for different weight fraction at different energy.

Figure 9 shows the MFP values of free silicone rubber, 20% micro—(Bi_2_O_3_, CdO, and SnO_2_)/SR, 20% nano—(Bi_2_O_3_, CdO, and SnO_2_)/SR, 50% micro—(Bi_2_O_3_, CdO, and SnO_2_)/SR and 50% nano—(Bi_2_O_3_, CdO, and SnO_2_)/SR composites as function of energy from 59.54 to 1408.01 keV. From MFP values, free silicone rubber has the higher MEP value, as free silicone rubber has a lower shielding ability. Moreover, nano—(Bi_2_O_3_, CdO, and SnO_2_)/SR composites have a lower value than micro—(Bi_2_O_3_, CdO, and SnO_2_)/SR composites at the same concentration. That’s illustrated by the fact that nanocomposites have a lower distance between two successive collisions which tends to improve shielding characterization than micro composites.

**Figure 10:**
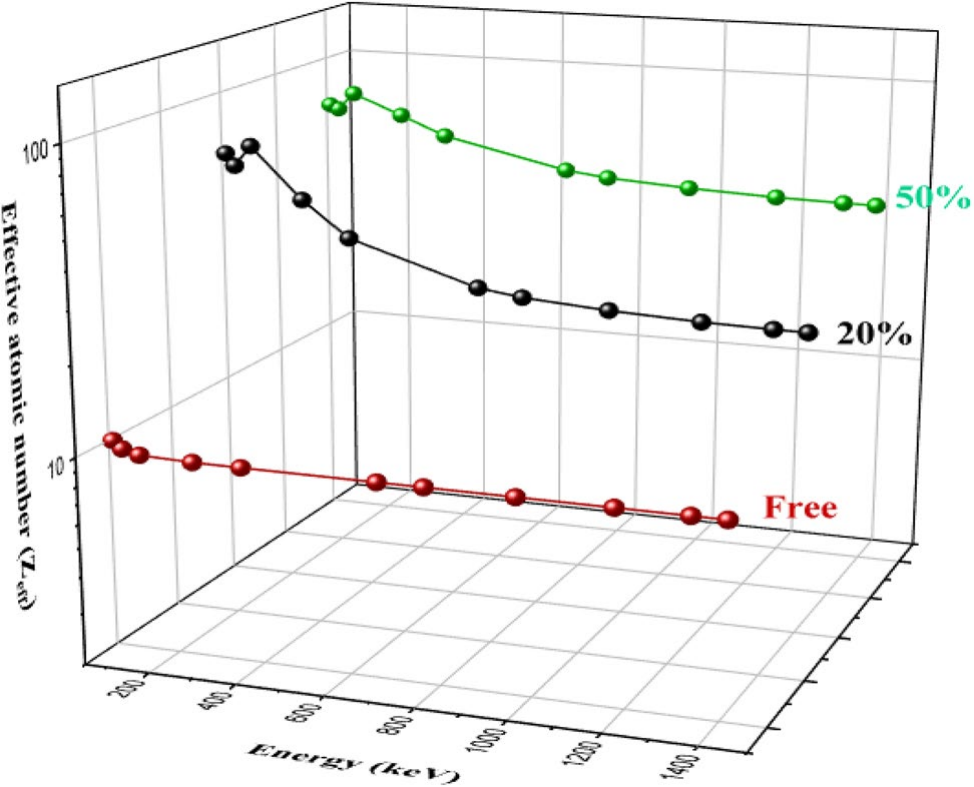
The effective atomic number of silicone rubber, micro-and nano—Bi_2_O_3_, CdO, and SnO_2_/SR for different weight fractions at different energy.

Figure 10 describes the Z_eff_ for silicone rubber, 20% Bi_2_O_3_, CdO, and SnO_2_/SR, and 50% Bi_2_O_3_, CdO, and SnO_2_/SR. Z_eff_ lines describe the attenuation ability of composites, which depends on the energy of the beam and on the Z of the elements in composites. It shows that the 50% Bi_2_O_3_, CdO, and SnO_2_/SR composite has the highest Z_eff_, which is reinforced with 50% Bi_2_O_3_, CdO, and SnO_2,_ while the silicone rubber composite has the lowest Z_eff_. The probability of interaction between beam photons and material depends on Z, where the photoelectric effect is directly proportional to Z^4^, Compton scattering depends on Z, and pair production interaction is influenced by Z^2^. So as the weight fraction of the high Z filler “Bi_2_O_3_, CdO, and SnO_2_” increases, the Z_eff_ increases, and attenuation increases.

Table 3 illustrates a comparison between recently published data and our current research on the improvement of attenuation properties using nanocomposites for gamma ray applications. According to the findings presented in Table 3, the utilization of nanoparticles significantly improves the attenuation properties. In the current study, the incorporation of nano—Bi_2_O_3_, CdO, and SnO_2_/SR composition results in an impressive attenuation of 33.58% for low energy and 16.47% for high energy. This remarkable enhancement can be attributed to the high density of the composite (2.122 g/cm^3^).

**Table 3:** Comparison of recent data on the enhancement of attenuation properties with nanocomposites for gamma ray**.**

References	Additive material	Weight fraction	Base matrix	Simulation method	Energy (keV)	Attenuation enhanced %
Malekzadeh et al.^15^	Bi	10%	Silicone	Experimental	60	17.5
					80	11.3
					100	10.9
Alharshan et al.^24^	CdO, PbO	30%	HDPE	Experimental	59.53	25.76
					80.99	25.27
					121.78	24.49
					244.69	23.85
					344.28	23.22
					356.01	22.96
					661.66	22.07
					778.9	21.76
					964.13	21.00
					1173.23	20.36
					1332.5	19.58
					1408.01	19.08
Gouda et al.^37^	SnO_2_	50%	Silicone rubber	Experimental	59.54	23.159
					80.99	23.470
					121.78	21.680
					244.69	21.047
					356.01	16.408
					661.66	18.294
					778.9	16.345
					964.13	16.013
					1173.23	11.826
					1332.5	13.734
					1408.01	12.411
El-khatib et al.^38^	CdO	40%	HDPE	Experimental	59.53	16.73
					80.99	16.17
					121.78	15.36
					244.69	14.72
					344.28	14.29
					356.01	14.16
					661.66	13.60
					778.9	12.65
					964.13	11.97
					1173.23	11.50
					1332.5	10.86
					1408.01	10.51
Present work	Bi_2_O_3_, CdO, and SnO_2_	50%	Silicone rubber	Experimental	59.54	33.58
					80.99	31.03
					121.78	29.02
					244.69	27.78
					356.01	26.61
					661.66	24.65
					778.9	22.81
					964.13	20.25
					1173.23	19.18
					1332.5	18.32
					1408.01	16.47

## Conclusion

This study aims to investigate the impact of particle size and weight fraction of micro and nano (Bi_2_O_3_, CdO, and SnO_2_) on the linear and mass attenuation coefficient of the metal oxide/SR composite at various photon energies. By measuring these coefficients, we can gain valuable insights into the behavior of the composite material. By employing advanced techniques such as SEM and TEM to examine the morphology of the composites and filler materials, the study reveals that nano composites exhibit a more uniform morphology compared to micro composites. This finding suggests that nano composites possess superior effectiveness in shielding against radiation. Furthermore, the study highlights the significance of tailoring composites with appropriate properties for efficient shielding by demonstrating that the size and concentration of filler materials impact the density of the composites. As the density of composites increases, their ability to attenuate radiation also increases. This observation underscores the importance of creating composites with optimal density to maximize their shielding capabilities. Moreover, this study delves into the impact of changes in the weight fraction of fillers on the tensile parameters of the composites, providing valuable insights into the mechanical properties of these materials. By understanding how variations in the weight fraction of fillers affect the tensile parameters, researchers and engineers can make informed decisions when designing and utilizing these composites.

## Data Availability

All data generated or analyzed during this study are included in this published article.
